# The Popular Science Monthly

**Published:** 1872-06

**Authors:** 


THE POPULAL SCIENCE MONTHLY. Messrs Appleton & Co. have commenced the publication of a new monthly under the above name. The magazine will be conducted by Prof. E L. Youmans, which fact alone will give assurance of its value. Among the list of contributors to the first number we notice the names of Herbert Spencer, R. G. Proctor, and others of scientific note. We have not had an opportunity as yet of seeing the Journal, but from what we hear of its worth wish it all success.

The late editors of the *National Medical Journal* announce that they will soon commence the publication of a new medical Journal called the "*Washington Medical Monthly.*"

Our valuable cotemporary and exchange the "*Journal of the Gynæcological Society*" has come to us since January with sixteen pages additional of interesting reading matter.

